# Correlations between APOE4 allele and regional amyloid and tau burdens in cognitively normal older individuals

**DOI:** 10.1038/s41598-022-18325-2

**Published:** 2022-08-22

**Authors:** Yun Jeong Hong, Chan-Mi Kim, Jae Hong Lee, Jorge Sepulcre

**Affiliations:** 1 Department of Neurology, Uijeongbu St. Mary’s Hospital, Catholic University of Korea, Seoul, Korea; 2 Gordon Center for Medical Imaging, Department of Radiology, Massachusetts General Hospital, Harvard Medical School, Boston, MA USA; 3 Athinoula A. Martinos Center for Biomedical Imaging, Department of Radiology, Massachusetts General Hospital, Harvard Medical School, 13th Street, Charlestown, MA 02129 USA; 4 Department of Neurology, Asan Medical Center, University of Ulsan College of Medicine, Seoul, Korea

**Keywords:** Neuroscience, Biomarkers, Medical research, Neurology

## Abstract

The correlations between apolipoprotein epsilon 4 (APOE4) status and regional amyloid, tau, and cortical thickness in cognitively normal elderly are not fully understood. Our cross-sectional study aimed to compare regional amyloid/tau burden, and cortical thickness according to APOE4 carrier status and assess correlations between APOE4 and Alzheimer’s disease (AD)-related biomarker burdens. We analyzed 185 cognitively normal participants from the Alzheimer’s Disease Neuroimaging Initiative (ADNI) cohort. Participants aged 55–90 with normal cognitive function were divided into amyloid ß-positive (Aß+) APOE4 carriers (group 1, n = 27), Aß+ APOE4 non-carriers (group 2, n = 29), and Aß− normal controls (group 0, n = 129). We compared amyloid depositions, tau depositions, and cortical thickness among the three groups and assessed correlations between APOE4 existence and imaging biomarkers adjusted for age and sex. The participants in group 2 were older than those in the other groups. The regional amyloid/tau standardized uptake value ratios (SUVRs) did not differ between groups 1 and 2, but the amyloid/tau SUVRs in most regions were numerically higher after adjusting for age difference. APOE4 allele had robust correlations with increased amyloid burden in the fronto-temporo-parietal cortical areas after adjustment for age and sex, but it had weaker and mixed correlations with the regional tau burden and did not have significant correlation with cortical thickness. We identified that the presence of APOE4 allele might be more highly associated with amyloid deposition than with other AD-related biomarkers such as tau or cortical thickness in cognitively normal elderly.

## Introduction

The apolipoprotein epsilon 4 (APOE4) allele is a well-known susceptibility gene for late-onset Alzheimer’s disease (AD)^1^. Presence of the APOE4 allele is associated with a higher risk of dementia and earlier age of AD onset in a dose-dependent manner maybe through an increase in the cerebral amyloid burden^2–4^. According to previous studies, the APOE4 allele might also be related to neuroinflammation, neurodegeneration, and pathologic tau deposition^5,6^.

Amyloid plaques and neurofibrillary tangles are key pathologic markers of AD, and in vivo quantification of the amyloid and tau burdens is possible using positron emission tomography (PET)^7^. The amyloid and tau burdens shown with PET imaging can identify preclinical AD, clarify relationships between biomarkers, and accelerate clinical trials as appropriate surrogate markers in the early stages of AD. However, despite of many previous studies investigating the role of APOE4 that confers AD risk^8^, fewer studies assessed the effects of APOE4 on the pathological manifestations of the disease; correlations between APOE4 allele and regional amyloid, tau, and neurodegenerative burdens in cognitively normal elderly people are not fully understood, and the few previous studies have reported inconsistent results^9–13^. Associations between APOE4 and amyloid depositions have been consistently reported, however, APOE4 effects on tau burdens are somewhat inconsistent^8–13^. While a previous study reported that APOE4 allele has different predispositions on amyloid/ tau burdens according to aging^11^, others showed independent effects of APOE4 allele on AD pathologies irrespective of aging^12,13^. Moreover, associations between APOE4 and regional amyloid/tau/neurodegenerative changes are not studied in cognitively normal participants^8–13^. Thus, the further studies are needed to understand the relationship between APOE4 allele existence and regional amyloid and tau accumulations, or even in cortical thickness, in cognitively normal subjects, and to clarify how amyloid status affects those relationships.

In this cross-sectional study, we assessed the correlations between APOE4 carrier status and regional AD-related biomarkers (amyloid burden, tau burden, and cortical thickness) in cognitively normal participants. We compared the demographics, clinical characteristics, regional amyloid/tau burdens using PET and cerebrospinal fluid (CSF), and cortical thickness of amyloid ꞵ-positive (Aß+) APOE4 carriers, Aß+ APOE4 non-carriers, and Aß− normal controls.

## Methods

### Participants

Our participants were part of the Alzheimer’s Disease Neuroimaging Initiative (ADNI)-3 longitudinal multicenter cohort study starting in 2016. Data used in the preparation of this study were obtained from the ADNI database (http://adni.loni.usc.edu/). We included 185 cognitively normal participants who underwent amyloid PET with F18-florbetapir, tau PET with F18-flortaucipir, magnetic resonance imaging (MRI), and APOE4 genotyping as part of the ADNI 3 cohort study. We excluded 4 Aβ + participants whose APOE4 carrier status was unknown. All participants were determined to be cognitively normal through a consensus diagnosis based on quantitative data and clinical and cognitive assessments by physicians, neuropsychologists, and study coordinators. In brief, the study inclusion criteria for cognitively normal participants were as follows: (1) aged 55–90, (2) normal function in the logical memory II delayed recall test after adjustment for educational level, (3) Mini-Mental State Examination (MMSE)^14^ score between 24 and 30 (educational level below 8 years can be permitted if the MMSE score is in the normal range), (4) clinical dementia rating (CDR)^15^ score of 0, (5) normal in both cognitive scores and activities of daily living (ADL) score, (6) geriatric depression scale score < 6, and (7) modified Hachinski ischemic score ≤ 4 (for further information, http://adni.loni.usc.edu/). We divided the participants into amyloid PET positive-APOE4 carriers (group 1, Aß+ APOE4+, n = 27), amyloid PET positive-APOE4 non-carriers (group 2, Aß+ APOE4−, n = 29), and an amyloid PET negative control group regardless of APOE4 carrier status (group 0, Aß−, n = 129).

The clinical characteristics, including neuropsychological tests results, and CSF biomarker data were obtained from the ADNI 3 files. Complete details of the methods adopted by ADNI 3 can be accessed at http://adni.loni.usc.edu/data-samples/clinical-data/. Aβ42, total tau, and phosphorylated tau (p-tau)181 values in the CSF samples were compared among the groups. The ADNI study was approved by the institutional review boards of all of the participating centers. Informed written consent was obtained from all participants at each center. We used ADNI data without any access to personal identifiable information.

### Neuropsychological tests

Cognitive/clinical assessments including neuropsychological tests were performed within 2 weeks before and after the imaging and biofluid collections. Cognitive tests included Alzheimer’s Disease Assessment Scale-Cognitive (ADAS-Cog13)^16^, MMSE, Montreal Cognitive Assessment (MoCA)^17^, and Rey Auditory Verbal Learning Test (RAVLT)^18^ for verbal memory functions. Global, function, and behavioral evaluations included CDR for global functions, Functional Assessment Questionnaire (FAQ)^19^ for functional evaluations, Neuropsychiatric Inventory (NPI)^20^ for behavioral evaluations, and Cognitive Change Index (CCI)^21^ for subjective cognitive declines.

### Neuroimaging

T1-weighted MRI was performed using an accelerated sagittal magnetization-prepared rapid gradient echo (MPRAGE) sequence on a 3-Tesla Siemens system or 3-Tesla Phillips system. Some T1-weighted MR images were acquired using an accelerated sagittal inversion recovery-prepared fast spoiled gradient-echo (IR-FSPGR) sequence on a 3-Tesla GE system.

Florbetapir (FBP) PET scanning was performed for 20 min in 4-by-5-min frames beginning 50–70 min after injection of 10.0 ± 1.0 mCi F18-FBP. Tau PET (^18^F-AV-1451; flortaucipir) scanning was performed for 30 min in 6-by-5-min frames beginning 75–105 min after injection of 10.0 ± 1.0 mCi F18-flortaucipir. The detailed PET acquisition methods are described at http://adni.loni.usc.edu/data-samples/pet/. All PET images were reconstructed using methods suitable for their scanner types.

### Neuroimaging analysis

Full information about how we preprocessed the MRI and PET data is provided in the Supplementary Data 1. In thickness, and segment region-of-interest (ROI) volumes^22,23^. We used the automated anatomical labeling (AAL) template^24^ to define the ROIs in this study. The ROIs were used to calculate the standard uptake value ratio (SUVR), perform partial volume correction (PVC), and measure regional PET uptakes. AAL ROIs segmented from individual MRIs were registered to individual PET data and normalized by the mean value in the cerebellar gray reference region to calculate SUVR^25^. PVC was performed in all PET images.

We checked amyloid-positivity across all participants using the recommended threshold of global FBP-SUVR ≥ 1.10 in each individual^26^. The global FBP-SUVR was calculated as the ratio of the mean FBP-SUVR in 6 cortical ROIs (frontal, temporal, precuneus, parietal, anterior cingulate, and posterior cingulate cortices) to that of the whole cerebellum reference region without performing PVC. We also measured the regional mean values of FBP-SUVR and tau-SUVR within 90 AAL ROIs in each individual for further ROI-based group analysis. To assess AD-related regional differences, we calculated the mean SUVRs of 10 regions (medial temporal, inferior temporal, lateral temporal, lateral frontal, inferior frontal, lateral parietal, precuneus, posterior cingulate, occipital, and striatum) in the 90 ROI-based SUVRs for each individual.

### Vertex-wise group comparisons

Surface-mapped FBP-SUVRs, tau-SUVRs, and cortical thicknesses were compared among groups after controlling for age and sex using a general linear model (GLM) in the SurfStat toolbox within MATLAB. We performed false discovery rate (FDR) correction, set at p < 0.05, in all vertex-wise group analyses for multiple comparisons^27^.

### Point-biserial correlation analysis between APOE4 status and imaging-based biomarkers

To examine associations between APOE4 status and regional amyloid, tau burdens, and cortical thickness, we performed a point-biserial correlation analysis, which measures the correlations between a binary variable and continuous variables. In this case, we analyzed the correlations between a categorical variable (APOE4 carriers = 1, APOE4 non-carriers = 0) and the surface-mapped imaging variables in the all cognitively normal participants, both Aβ-positive and Aβ-negative groups.

### Statistical analysis

We compared regional amyloid burden, regional tau burden, regional cortical thickness, neuropsychological tests results, and CSF biomarkers including CSF Aβ42 and tau values among the groups using one-way analysis of variance (ANOVA) with multiple comparisons and post-hoc Bonferroni corrections for continuous variables and chi square tests for categorical variables. For group comparisons of regional amyloid and tau SUVRs, we used an analysis of covariance (ANCOVA) methods adjusted for age. In addition, to clarify effects of APOE4 on tau depositions or cortical thickness through Aβ-independent pathways, we compared regional amyloid, tau, and cortical thickness between APOE4 carriers and APOE4 non-carriers. It was conducted in two ways; model 1 (after controlling for age, sex, and global amyloid burdens) and model 2 (after controlling for age and sex).

Statistical analyses were performed using SPSS 19.0 (SPSS, Chicago, IL, USA) software. Statistical significance was defined as *p* < 0.05, and all tests were 2-sided.

### Ethics approval and consent to participate

The study protocol and informed consent form were approved by the institutional review boards of all of the participating centers. Informed written consent was obtained from all participants at each center. The study was conducted in accordance with the Declaration of Helsinki and principles of Good Clinical Practice.

## Results

### Baseline characteristics

The study population flowchart is shown in Supplementary Fig. 1. The participants (n = 185) were divided into 3 groups: Group 1 (n = 27, Aβ + APOE4+), Group 2 (n = 29, Aβ + APOE4−), and Group 0 (n = 129, Aβ−). In the group 0, 24.35% (28/115) were APOE4 carriers. The baseline demographics except the age were similar among the groups (Table 1). The participants in group 2 were older than those in the other groups (group 0, group 1). The global amyloid SUVR values were higher in the Aβ+ groups (group 1, 2) than in the Aβ- group (group 0), whereas the global tau SUVR values were similar among the groups. Tau PET positive (global SUVR > 1.25) participants were only 5 (3 in the Aꞵ+ groups and 2 in the Aꞵ− group). The CSF Aβ42 and tau values differed between the Aβ-positive groups (group 1, 2) and Aβ-negative group (group 0). Group 1 had the lowest CSF Aβ42 level, but the numerical difference between groups 1 and 2 was not statistically significant (Table 1). Although the general cognitive scores (MMSE, MOCA) were similar among the groups, the Rey auditory verbal learning test (RAVLT) forgetting scores were lower in the Aβ+ groups than the Aβ− group, and a few tests including ADAS-cog13 total scores, FAQ total score, and RAVLT immediate recall and learning function showed worse scores in group 2 than in the other groups (group 0, 1).

**Table 1:** Clinical and demographic characteristics according to the biomarker findings.

Variables	Group 0 (Aβ−) (n = 129)	Group 1 (Aβ+ APOE4+) (n = 27)	Group 2 (Aβ+ APOE4−) (n = 29)	*P* value	Post-hoc
Age	72.70 ± 9.81	75.04 ± 7.19	82.14 ± 5.89	< 0.001*	2 > 1 = 0
Female n, %	74 (57.36%)	17 (62.96%)	18 (62.07%)	0.806	
Education, yrs	16.45 ± 3.21	16.11 ± 3.92	15.28 ± 3.83	0.247	1 = 2 = 0
APOE4 allele, 0/1/2, n (%)	87/24/4 (75.7/20.9/3.5)	0/24/3 (0/88.9/11.1)	29/0/0 (100/0/0)	< 0.001*	
Global SUVR, amyloid	0.975 ± 0.057	1.280 ± 0.165	1.298 ± 0.172	< 0.001*	1 = 2 > 0
Global SUVR, tau	1.040 ± 0.073	1.066 ± 0.066	1.076 ± 0.078	0.028*	1 = 2 = 0
CSF Aβ42	1520.54 ± 695.06	691.94 ± 188.56	1017.52 ± 550.63	< 0.001*	1 = 2 < 0
CSF Aβ40	18,686.22 ± 5485.07	18,593.75 ± 5202.44	19,971.25 ± 5111.82	0.451	1 = 2 = 0
CSF p tau 181	18.24 ± 6.84	27.16 ± 10.09	27.61 ± 12.43	< 0.001*	1 = 2 > 0
CSF t tau	214.79 ± 67.62	290.33 ± 93.81	294.88 ± 103.64	< 0.001*	1 = 2 > 0
MMSE score	28.87 ± 2.81	28.74 ± 1.72	28.55 ± 1.43	0.821	1 = 2 = 0
MOCA score	25.74 ± 4.38	24.59 ± 5.81	25.24 ± 2.50	0.445	1 = 2 = 0
ADAS-cog 13	12.53 ± 4.83	12.93 ± 5.70	15.44 ± 5.41	0.045*	2 > 0 = 1
ADAS-Q1 immediate recall	2.79 ± 1.25	2.67 ± 1.43	3.33 ± 1.34	0.089	1 = 2 = 0
ADAS-Q3 visuospatial	0.34 ± 0.52	0.30 ± 0.54	0.48 ± 0.51	0.342	1 = 2 = 0
ADAS-Q4 delayed recall	2.69 ± 2.03	2.37 ± 1.90	3.41 ± 2.47	0.144	1 = 2 = 0
ADAS-Q5 naming	0.05 ± 0.28	0.04 ± 0.19	0.07 ± 0.26	0.889	1 = 2 = 0
ADAS-Q8 recognition	5.58 ± 1.53	5.52 ± 1.91	5.93 ± 1.53	0.527	1 = 2 = 0
FAQ_total	0.23 ± 1.39	0.19 ± 0.56	1.79 ± 5.15	0.005*	2 > 0 = 1
RAVLT_forgetting (trial 5-delayed)	1.40 ± 2.31	2.15 ± 3.01	3.17 ± 3.06	0.003*	1 = 2 > 0
RAVLT_immediate recall	18.74 ± 23.48	16.26 ± 22.43	32.07 ± 18.79	0.011*	2 > 0 = 1
RAVLT_learning	2.20 ± 3.08	2.52 ± 3.59	3.83 ± 2.90	0.044*	1 = 2 > 0 = 1
NPI_total	1.24 ± 2.92	1.89 ± 4.59	1.48 ± 3.42	0.636	1 = 2 = 0
CCI_total	27.83 ± 7.29	29.41 ± 8.22	27.33 ± 6.12	0.753	1 = 2 = 3

*Aβ* amyloid β-positive, *APOE* apolipoprotein epsilon, *CSF* cerebrospinal fluid, *SUVR* standardized uptake value ratio, *MMSE* Mini-Mental State Examination, *MOCA* Montreal cognitive assessment, *ADAS-cog* Alzheimer’s disease assessment scale-cognitive subscale, *FAQ* functional activities questionnaire, *RAVLT* Rey auditory verbal learning test, *NPI* neuropsychiatric inventory, *CCI* cognitive change index.

Mean ± SD. *p ≤ 0.05.

### Regional amyloid and tau burdens

**Figure 1:**
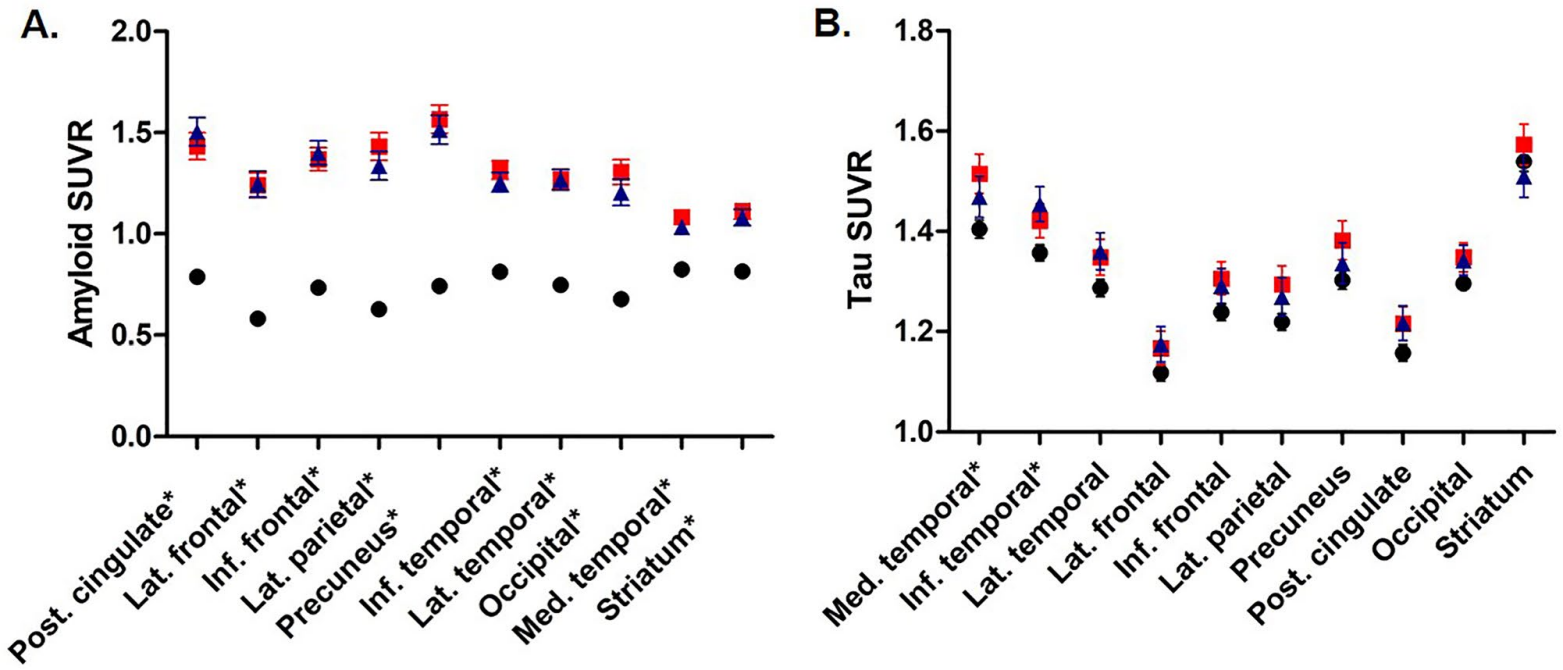
Regional amyloid and tau burdens (left: amyloid SUVR, right: tau SUVR). Mean ± standard deviations. *Significant difference between the groups. Circle (black): group 0 (Aß−); square (red): group 1 (Aß+ APOE4+); triangle (blue): group 2 (Aß+ APOE4−). (**A**) Regional amyloid SUVR adjusted for age; (**B**) regional tau SUVR adjusted for age.

Regarding regional amyloid SUVR values, the Aβ+ groups (group 1, 2) showed higher amyloid SUVRs than the Aβ− group in all regions. The amyloid SUVRs did not differ statistically between group 1 and 2, although those in group 1 were numerically higher than those in group 2 in most regions after adjusting for age (Supplementary Table 1, Fig. 1A). Regionally, the amyloid SUVRs in the precuneus and posterior cingulate were higher than those in other regions (Fig. 1A).

Regarding regional tau SUVR values, the Aβ+ groups (group 1, 2) showed higher tau SUVRs than the Aβ− group in medial and inferior temporal lobes (Supplementary Table 1). The medial temporal tau SUVRs of group 1 were significantly higher than those of group 0, and the inferior temporal tau SUVRs of group 2 were significantly higher than those of group 0 after adjusting for age (Fig. 1B). The tau burdens did not differ statistically between group 1 and 2, although the SUVRs in the medial temporal, inferior frontal, lateral parietal, and precuneus of group 1 were numerically higher than those in group 2; especially the SUVRs in the medial temporal lobes showed trends of difference after adjusting for age (Fig. 1B). Regionally, the tau SUVRs showed variabilities in regional depositions; the medial temporal lobes were numerically higher than those in other regions (Fig. 1B).

Comparing regional amyloid, tau, and cortical thickness between APOE4 carriers and non-carriers that were conducted in model 1 (after controlling for age, sex, and global amyloid burdens) and model 2 (after controlling for age and sex) showed no significant group difference in any regions (Supplementary Tables 2, 3).

### Vertex-wise group comparisons

The vertex-wise group comparisons of the amyloid SUVRs, tau SUVRs, and cortical thickness are shown in Supplementary Fig. 2. The amyloid SUVRs of the Aβ+ groups were higher in diffuse cortical regions compared with those of the Aβ− group (Supplementary Fig. 2A), whereas the tau SUVRs of the Aβ+ groups were higher only in parts of the temporal and frontal lobes and even smaller parts of the parietal cortex. Cortical thickness did not show group differences (Supplementary Fig. 2A). Group 1 showed only a trend of greater tau deposition in the temporal lobes (medial, inferior, and lateral temporal cortex) and parts of the frontal lobes compared with group 0 (Supplementary Fig. 2B). Group 2 showed significantly greater tau deposition in parts of the lateral temporal and posterior parietal lobes compared to group 0 (Supplementary Fig. 2C). Groups 1 and 2 did not differ significantly in any of the vertex-wise comparisons (Supplementary Fig. 2D). Cortical thickness did not show any group differences, although group 2 showed a trend of greater cortical thinning in parts of the frontal, parietal, and temporal cortical areas compared with group 0 (Supplementary Fig. 2C).

### Correlations between APOE4 and AD biomarkers

**Figure 2:**
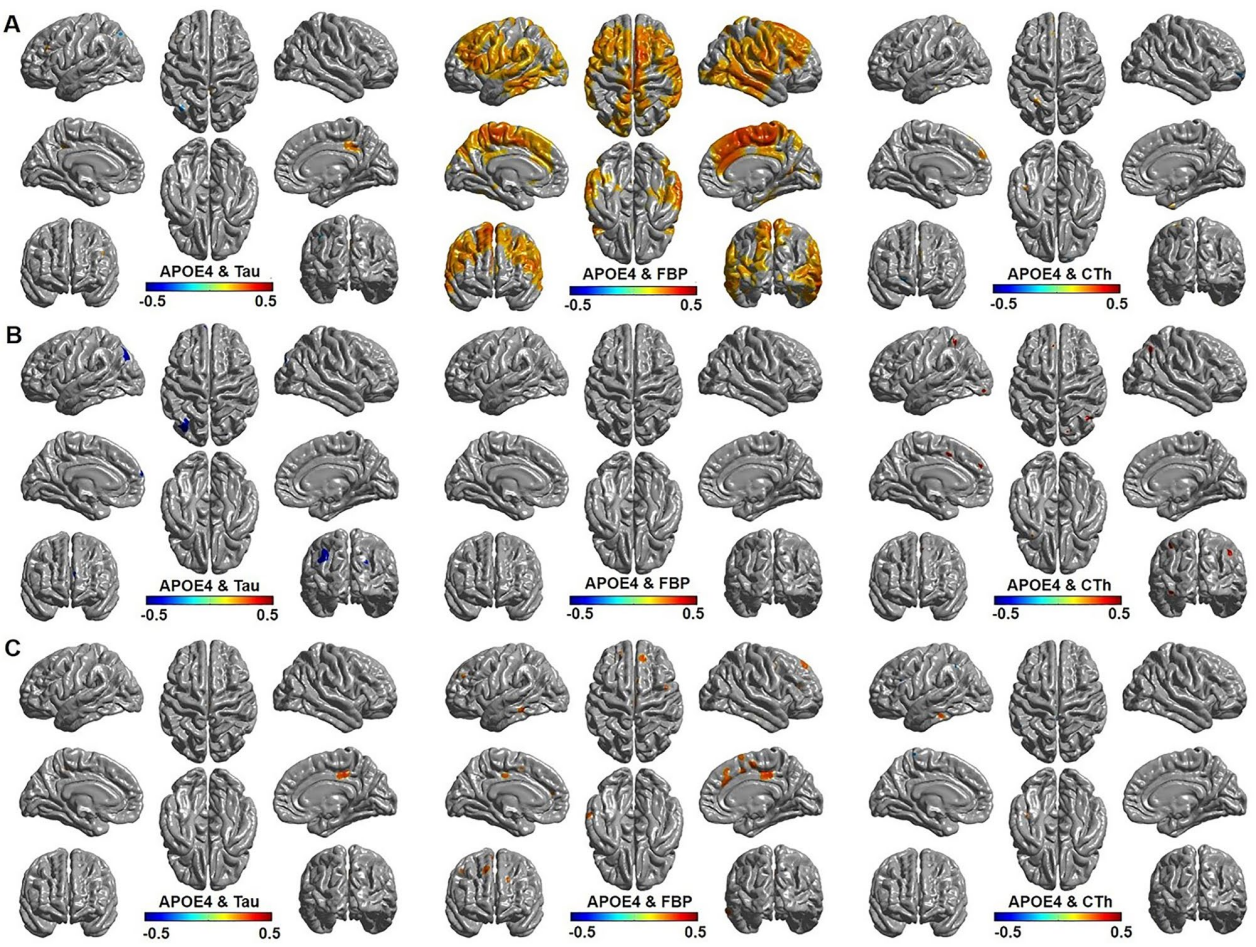
Vertex-wise point biserial correlation analysis between APOE4 status (APOE4 carrier = 1, non-carrier = 0) and the variables (tau, amyloid, and cortical thickness controlling for age and sex by FDR corrected *p* < 0.05). (**A**) Correlation between APOE4 and biomarkers in all cognitive normal participants; (**B**) Correlation between APOE4 and biomarkers in cognitive normal Aβ+ participants; (**C**) Correlation between APOE4 and biomarkers in cognitive normal Aβ− participants. Tau burden (left), amyloid burden (mid), and cortical thickness (right).

Association analysis between APOE4 and the amyloid/tau burdens is shown in Fig. 2. The amyloid burden was strongly associated with APOE4 carrier status in the frontal, lateral temporal, and parietal cortices in all cognitively normal participants (Fig. 2A). Tau burden showed mixed results; tau depositions in the posterior cingulate were positively associated and tau depositions in parts of lateral parietal cortex were negatively associated with APOE4 existence in all participants. Cortical thickness was not significantly associated with APOE4 carrier status except a small part of medial frontal cortex that shows positive association with APOE4 allele (Fig. 2A). When assessing only in Aβ+ participants (Fig. 2B), parts of lateral parietal cortex showed negative associations between APOE4 and tau and parts of lateral parietal and medial frontal cortex showed positive associations between APOE4 and cortical thickness. Amyloid burden did not show significant association with APOE4 allele when assessing only in Aβ+ participants. When assessing in Aβ− participants (Fig. 2C), amyloid burdens showed positive associations in the multiple fronto-temporal cortex, tau burdens in the posterior cingulate, and cortical thickness in a part of inferior temporal cortex with APOE4 existence.

## Discussion

In this study, we compared the clinical characteristics, regional amyloid and tau depositions, and cortical thickness among Aß+ APOE4 carriers, Aß+ APOE4 non-carriers, and normal controls to assess correlations between the APOE4 allele and AD-related biomarker burdens in cognitively normal elderly people.

Our study showed 3 major findings. First, APOE4 carrier status had robust correlations with increased amyloid burden in the fronto-temporo-parietal cortical areas in cognitively normal participants, both Aß+ and Aß−. While this status had weaker and mixed correlations with the regional tau in our study. APOE4 allele showed positive correlations with cortical thickness in small parts of fronto-parietal cortex. The presence of APOE4 allele might be more highly associated with amyloid deposition than with other AD-related biomarkers, such as tau or cortical thickness, and thus contribute to vigorous amyloidosis in cognitively normal elderly people. Low CSF Aß42 level might also explain why the Aß+ APOE4 carriers had more amyloidosis. While the presence of APOE4 did not correlate with any regional amyloid burden when we assessed only Aß+ participants, APOE4 still correlated with parts of fronto-temporo-parietal cortex when we assessed only Aß− participants. However, we did not show any differences in regional amyloid or tau burden between Aß+ APOE4+ and Aß+ APOE4− participants. The lack of statistically significant differences between the two groups can be explained as follows: (1) Aß status is an overlapping and even more relevant factor in AD-related biomarkers than is the APOE4 allele, and (2) the age difference between the Aß+ APOE4 carrier group and Aß+ APOE4 non-carrier group should be considered because aging is an important factor in AD-related biomarkers^28–30^. A previous study also reported that tau SUVRs were associated with age in many regions especially the medial temporal lobes^8,29^. Because the Aß+ APOE4− participants were older, they might have influenced more amyloid and tau burdens and cortical thinning, attenuating the impacts of the APOE4 allele. Regarding the weak correlations between APOE4 allele and tau burdens, only a few in this population revealed tau PET-positivity and most participants with normal cognition had limited focal tau depositions mainly in the medial temporal lobes could be another explanation. Small parts of posterior cingulate cortex showed positive correlations and lateral parietal cortex showed negative correlations with APOE4 allele, suggesting mixed impacts on tau burdens according to APOE4 carrier status. Our results also showed similar trends to the previous AD studies that high tau depositions in medial temporal region in APOE4 + AD participants, although it did not reach statistical significance. A previous systematic review reported that APOE4 + AD patients showed relatively more tau in the medial temporal lobe, while APOE4 − AD patients had more tau in other cortical regions, such as the frontal and parietal lobes^8^. Our mixed results between APOE4 and tau depositions could be suggestive of different tau depositions according to APOE4 existence in the cognitively normal participants.

Second, the Aß+ groups showed increased amyloid deposition in diffuse cortical areas and increased tau deposition in the regional temporal lobes, even though our entire sample was cognitively normal. Tau deposition has regional variabilities which shows different pattern from diffuse amyloid depositions in the Aß+ groups. Regionally, the medial, inferior, and lateral temporal cortex revealed relatively higher tau burdens than other regions, as predicted by the Braak neurofibrillary tangles stages^31^. Moreover, the medial temporal tau burden was higher in the Aß+ APOE4+ group than in the control group. Intriguingly, we found increased regional tau burdens in the inferior temporal, lateral temporal, inferior frontal, and parietal cortices despite normal cognition, which is consistent with a previous study that reported that the tau burden was increased in widespread parts of the medial temporal and extra-temporal regions of cognitively unimpaired participants^29^. Those authors explained their findings by widespread primary age-related tauopathy (PART) in the brain, although its biological significance and clinical importance have not been clarified^29^.

Third, we found that groups 1 and 2 had different characteristics. Group 1 showed the lowest CSF Aß42 level, suggesting vigorous amyloidosis and comparable amyloid/tau burdens with group 2, despite the younger age of group 1. The participants in group 2 were older and showed trends of greater cortical thinning in the fronto-temporo-parietal cortex and significant inferior temporal tau deposition compared to the normal controls, after adjusting for age. Although the differences with group 1 did not reach statistical significance, tau deposition and cortical thinning seemed to be more prominent, and the neurodegeneration might be caused by a combination of aged-related and non-tau pathologies. The clinical significance of PART and non-tau pathologies should be further assessed using longitudinal data.

Our results should be interpreted with caution due to some limitations. First, the Aß+ APOE4− group was significantly older than other two groups. Because aging might be a strong factor related with amyloid, tau, and neurodegenerations, age difference between the groups might attenuate the influences of APOE4 allele on biomarker status although we statistically adjusted for age. Second, the absence of longitudinal follow up data would be another limitation. We could assess only cross-sectional associations between APOE4 existence and regional biomarker status. Rates of amyloid/tau depositions and future cognitive declines were not identified in the study. Further longitudinal studies with serial amyloid and tau PET imaging data would clarify impacts of APOE4 on AD-related biomarker trajectories in this population. Lastly, lack of other biomarkers, including cerebrovascular disease, α-synuclein, TAR DNA-binding protein 43 (TDP-43), or Lewy body, should be considered due to the fact that those contribute to neurodegenerations. Although there are a few limitations, this study included relatively large number of cognitively normal participants who underwent APOE genotyping, detailed neuropsychological tests, CSF study, FBP-PET, tau PET, and volumetric MRIs to identify correlations between APOE4 allele and biomarker findings.

## Conclusion

In preclinical AD, APOE4 carrier status had robust correlations with increased amyloid burden in diffuse cortical areas, while, it had weaker and mixed correlations with the regional tau burden and did not have correlation with the cortical thickness. The effects of APOE4 carrier status on clinical progression to dementia should be clarified in longitudinal studies.

## Supplementary Information

Supplementary Information 1.

Supplementary Table 1.

Supplementary Table 2.

Supplementary Table 3.

Supplementary Figure 1.

Supplementary Figure 2.

## Data Availability

Data used in preparation of this article were obtained from the ADNI database (adni.loni.usc.edu). As such, the investigators within the ADNI contributed to the design and implementation of ADNI and/or provided data but did not participate in analysis or writing of this report. A complete listing of ADNI investigators can be found at: http://adni.loni.usc.edu/wp-content/uploads/how_to_apply/ADNI_Acknowledgement_List.pdf. The datasets used and/or analyzed during the current study are available from the corresponding author on reasonable request.

## Abbreviations

APOE4: Apolipoprotein epsilon 4
AD: Alzheimer’s disease
PET: Positron emission tomography
CSF: Cerebrospinal fluid
Aß+: Amyloid β-positive
MMSE: Mini-mental state examination
CDR: Clinical dementia rating
ADL: Activities of daily living
p-tau: Phosphorylated tau
MPRAGE: Magnetization-prepared rapid gradient echo
IR-FSPGR: Inversion recovery-prepared fast spoiled gradient-echo
FBP: Florbetapir
ROI: Region-of-interest
AAL: Automated anatomical labeling
SUVR: Standard uptake value ratio
PVC: Partial volume correction
GLM: General linear model
FDR: False discovery rate
ANOVA: One-way analysis of variance
ANCOVA: Analysis of covariance
RAVLT: Rey auditory verbal learning test
TDP-43: TAR DNA-binding protein 43
