# Supplementary material for: Correlations between APOE4 allele and regional amyloid and tau burdens in cognitively normal older individuals
Source: Sci Rep. 2022 Aug 22;12:14307. doi: 10.1038/s41598-022-18325-2 (PMC9395408; doi:10.1038/s41598-022-18325-2)
Supplement: Supplementary file 1 — Supplementary Information 1. [file 41598_2022_18325_MOESM1_ESM.docx]

**Supplementary data (detailed methods for neuroimaging analyses)**

**MRI data preprocessing**

All T1-weighted MRIs were automatically processed by FreeSurfer using the recon-all procedure (FreeSurfer version 6.0.0; <http://surfer.nmr.mgh.harvard.edu/>) to reconstruct cortical surfaces, measure cortical thickness, and segment region-of-interest (ROI) volumes [1,2]. Gray matter (GM), white matter, and cerebrospinal fluid were segmented from the T1-MRIs and volume-registered into the Montreal Neurological Institute/International Consortium for Brain Mapping (MNI/ICBM) space using the surface deformation procedure [3]. We used the automated anatomical labeling (AAL) template [4] to define the ROIs in this study. The AAL template was inversely registered to each native T1-MRI using an inverse transformation matrix in each individual. The ROIs were used to calculate the standard uptake value ratio (SUVR), perform partial volume correction (PVC), and measure the regional PET uptakes in PET image processing. The native MRIs were automatically mapped onto a common surface template using a surface-based averaging technique considering cortical folding patterns, and cortical thickness was calculated by measuring the inner and outer surfaces via FreeSurfer.

**PET data preprocessing**

Both FBP PET and tau PET data were processed using the FMRIB Software Library (FSL; <http://fsl.fmrib.ox.ac.uk>) and the FreeSurfer PetSurfer procedure (FreeSurfer version 6.0.0; <http://surfer.nmr.mgh.harvard.edu/fswikiPetSurfer>) to perform co-registration, calculate SUVRs, and perform PVC [5]. Each individual FBP PET or tau PET image was co-registered to the corresponding native T1-weighted MRI using rigid-body registration with a mutual information cost function. AAL ROIs that segmented individual MRIs were inversely registered to each individual FBP PET or tau PET scan using an inverse transformation of each co-registration matrix. Then, each individual FBP PET or tau PET scan was normalized to the mean value in the cerebellar gray reference region to calculate the SUVR [6]. PVC was performed in all PET images using an extended Müller-Gärtner method in the PetSurfer procedure to estimate the true radioactivity concentration in a human brain (GM only) considering the heterogeneity of GM activity in a four-compartment model [7]. The GM threshold for PVC was set at 0.1, and the point spread function for PVC was estimated to be 8 mm. Then, individual PVC FBP-SUVR and tau-SUVR images were co-registered to the corresponding MRI and then the MNI/ICBM template using the transformation matrixes obtained from the previous step. The registered volume–based PVC PET SUVR images for each individual were sampled onto the cortical surface by mapping the value of the middle point between the inner and outer surfaces of each vertex point via the FreeSurfer procedure for a vertex-wise group analysis.

**References**

1. Dale AM, Fischl B, Sereno MI. Cortical surface-based analysis. I. Segmentation and surface reconstruction. Neuroimage. 1999;9(2):179-94.

2. Desikan RS, Ségonne F, Fischl B, Quinn BT, Dickerson BC, Blacker D, et al. An automated labeling system for subdividing the human cerebral cortex on MRI scans into gyral based regions of interest. Neuroimage. 2006;31(3):968-80.

3. Ségonne F, Dale AM, Busa E, Glessner M, Salat D, Hahn HK, et al. A hybrid approach to the skull stripping problem in MRI. Neuroimage. 2004;22(3):1060-75.

4. Tzourio-Mazoyer N, Landeau B, Papathanassiou D, Crivello F, Etard O, Delcroix N, et al. Automated anatomical labeling of activations in SPM using a macroscopic anatomical parcellation of the MNI MRI single-subject brain. Neuroimage. 2002;15(1):273-89.

5. Greve DN, Salat DH, Bowen SL, Izquierdo-Garcia D, Schultz AP, Catana C, et al. Different partial volume correction methods lead to different conclusions: An (18)F-FDG-PET study of aging. Neuroimage. 2016;132:334-43.

6. Sepulcre J, Grothe MJ, d'Oleire Uquillas F, Ortiz‐Teran L, Diez I, Yang HS, et al. Neurogenetic contributions to amyloid beta and tau spreading in the human cortex. Nat Med. 2018;24(12):1910-8.

7. Müller-Gärtner HW, Links JM, Prince JL, Bryan RN, McVeigh E, Leal JP, et al. Measurement of radiotracer concentration in brain gray matter using positron emission tomography: MRI-based correction for partial volume effects. J Cereb Blood Flow Metab. 1992;12(4):571-83

**Supplementary Fig. 1** Study flowchart

*APOE4* apolipoprotein epsilon 4

**Supplementary Fig. 2** Vertex-wise group comparisons of the biomarkers

Surface-based group differences were partial volume corrected while controlling for age, sex, and education. The color bars indicate the T-value, with warm colors indicating a high burden/cortical thickness and cool colors indicating low burden/cortical thinning.

A. Aβ+ vs. Aβ- (FBP SUVR (left; FDR corrected *p*<0.05), tau-SUVR (mid; FDR corrected *p*<0.05), and cortical thickness (right; uncorrected *p*<0.05)); B. Aβ+APOE4+ vs. Aβ- (FBP SUVR (left; FDR corrected *p*<0.05), tau-SUVR (mid; uncorrected *p*<0.05), and cortical thickness (CTh, right; uncorrected *p*<0.05)); C. Aβ+ APOE4- vs. Aβ- (FBP SUVR (left; FDR corrected *p*<0.05), tau-SUVR (mid; FDR corrected *p*<0.05), and cortical thickness (right; uncorrected *p*<0.05)); D. Aβ+APOE4+ vs. Aβ+APOE4- (FBP SUVR (left; uncorrected *p*<0.05), tau-SUVR (mid; uncorrected *p*<0.05), and cortical thickness (rt; uncorrected *p*<0.05))
