# Supplementary material for: Correlations between APOE4 allele and regional amyloid and tau burdens in cognitively normal older individuals
Source: Sci Rep. 2022 Aug 22;12:14307. doi: 10.1038/s41598-022-18325-2 (PMC9395408; doi:10.1038/s41598-022-18325-2)
Supplement: Supplementary file 2 — Supplementary Table 1. [file 41598_2022_18325_MOESM2_ESM.docx]

**Supplementary Table 1** Regional amyloid and tau burden (age-adjusted values, also shown in figure 2)

| Areas | Amyloid (-)  (G0,n=129) | Amyloid (+)  & APOE4+ (G1,n=27) | Amyloid (+)  & APOE4- (G2,n=29) | *P* value | Post-hoc |
| --- | --- | --- | --- | --- | --- |
| Amyloid_post cingulate | 0.787 ± 0.032 | 1.432 ± 0.067 | 1.504 ± 0.070 | < 0.001* | 1=2>0 |
| Amyloid_lateral frontal | 0.581 ± 0.029 | 1.239 ± 0.062 | 1.245 ± 0.065 | < 0.001* | 1=2>0 |
| Amyloid_inf frontal | 0.734 ± 0.027 | 1.368 ± 0.057 | 1.400 ± 0.059 | < 0.001* | 1=2>0 |
| Amyloid_lateral parietal | 0.627 ± 0.032 | 1.431 ± 0.068 | 1.336 ± 0.070 | < 0.001* | 1=2>0 |
| Amyloid_precuneus | 0.742 ± 0.033 | 1.566 ± 0.070 | 1.514 ± 0.072 | < 0.001* | 1=2>0 |
| Amyloid_inf temporal | 0.812 ± 0.022 | 1.315 ± 0.046 | 1.255 ± 0.048 | < 0.001* | 1=2>0 |
| Amyloid_lateral temporal | 0.748 ± 0.023 | 1.270 ± 0.049 | 1.267 ± 0.051 | < 0.001* | 1=2>0 |
| Amyloid_occipital | 0.678 ± 0.029 | 1.304 ± 0.062 | 1.204 ± 0.065 | < 0.001* | 1=2>0 |
| Amyloid_med temporal | 0.825 ± 0.016 | 1.083 ± 0.033 | 1.034 ± 0.035 | < 0.001* | 1=2>0 |
| Amyloid_striatum | 0.814 ± 0.018 | 1.112 ± 0.039 | 1.080 ± 0.040 | < 0.001* | 1=2>0 |
| Tau_med temporal | 1.404 ± 0.018 | 1.515 ± 0.039 | 1.469 ± 0.041 | 0.026* | 1=2>0=2 |
| Tau_inf temporal | 1.357 ± 0.016 | 1.421 ± 0.034 | 1.454 ± 0.035 | 0.024* | 1=2>0=1 |
| Tau_lateral.temporal | 1.287 ± 0.017 | 1.348 ± 0.036 | 1.360 ± 0.037 | 0.106 | 0=1=2 |
| Tau_lateral frontal | 1.118 ± 0.016 | 1.167 ± 0.034 | 1.175 ± 0.035 | 0.197 | 0=1=2 |
| Tau_inf frontal | 1.238 ± 0.016 | 1.306 ± 0.033 | 1.291 ± 0.035 | 0.113 | 0=1=2 |
| Tau_lateral parietal | 1.219 ± 0.017 | 1.294 ± 0.037 | 1.269 ± 0.039 | 0.139 | 0=1=2 |
| Tau_precuneus | 1.302 ± 0.018 | 1.382 ± 0.039 | 1.336 ± 0.041 | 0.176 | 0=1=2 |
| Tau_post cingulate | 1.158 ± 0.016 | 1.216 ± 0.034 | 1.217 ± 0.035 | 0.153 | 0=1=2 |
| Tau_occipital | 1.296 ± 0.014 | 1.348 ± 0.029 | 1.342 ± 0.030 | 0.162 | 0=1=2 |
| Tau_striatum | 1.539 ± 0.019 | 1.573 ± 0.041 | 1.510 ± 0.042 | 0.560 | 0=1=2 |
| CTh_paraphippocampal | 2.774 ± 0.019 | 2.786 ± 0.040 | 2.709 ± 0.042 | 0.331 | 0=1=2 |
| CTh_inf temporal | 2.689 ± 0.012 | 2.714 ± 0.026 | 2.689 ± 0.027 | 0.673 | 0=1=2 |
| CTh_lateral.temporal | 2.553 ± 0.010 | 2.571 ± 0.020 | 2.560 ± 0.021 | 0.705 | 0=1=2 |
| CTh_lateral frontal | 2.419 ± 0.010 | 2.436 ± 0.022 | 2.431 ± 0.022 | 0.720 | 0=1=2 |
| CTh_inf frontal | 2.503 ± 0.009 | 2.516 ± 0.019 | 2.518 ± 0.020 | 0.687 | 0=1=2 |
| CTh_lateral parietal | 2.294 ± 0.009 | 2.327 ± 0.020 | 2.298 ± 0.021 | 0.323 | 0=1=2 |
| CTh_precuneus | 2.314 ± 0.010 | 2.324 ± 0.022 | 2.286 ± 0.023 | 0.463 | 0=1=2 |
| CTh_post cingulate | 1.734 ± 0.012 | 1.716 ± 0.027 | 1.743 ± 0.028 | 0.764 | 0=1=2 |
| CTh_occipital | 2.133 ± 0.009 | 2.162 ± 0.018 | 2.138 ± 0.019 | 0.361 | 0=1=2 |

*Abbreviations*: *APOE4* apolipoprotein epsilon 4, *CTh* cortical thickness

Mean values ± standard errors. *Statistically significant.
