# Supplementary material for: Correlations between APOE4 allele and regional amyloid and tau burdens in cognitively normal older individuals
Source: Sci Rep. 2022 Aug 22;12:14307. doi: 10.1038/s41598-022-18325-2 (PMC9395408; doi:10.1038/s41598-022-18325-2)
Supplement: Supplementary file 3 — Supplementary Table 2. [file 41598_2022_18325_MOESM3_ESM.docx]

**Supplementary table 2** Regional amyloid, tau, and cortical thickness in APOE4 carriers and APOE4 non-carriers and their group differences after controlling for age, sex, and global amyloid burden

|  | Tau SUVRs | | Amyloid SUVRs | | Cortical thickness | | Tau | Amyloid | Cortical thickness |
| --- | --- | --- | --- | --- | --- | --- | --- | --- | --- |
| AAL ROIs | APOE4+ | APOE4- | APOE4+ | APOE4- | APOE4+ | APOE4- | adjusted *p* | adjusted *p* | adjusted *p* |
| Precentral_L | 1.049±0.172 | 1.033±0.207 | 0.870±0.421 | 0.746±0.426 | 2.456±0.166 | 2.422±0.153 | 0.620 | 0.349 | 0.946 |
| Precentral_R | 1.070±0.177 | 1.075±0.218 | 0.886±0.424 | 0.767±0.413 | 2.379±0.147 | 2.344±0.149 | 0.851 | 0.631 | 0.581 |
| Frontal_Sup_L | 1.035±0.225 | 0.997±0.250 | 0.776±0.526 | 0.591±0.533 | 2.494±0.121 | 2.472±0.139 | 0.322 | 0.828 | 0.601 |
| Frontal_Sup_R | 1.091±0.226 | 1.064±0.265 | 0.858±0.557 | 0.668±0.539 | 2.482±0.153 | 2.463±0.133 | 0.458 | 0.937 | 0.901 |
| Frontal_Sup_Orb_L | 1.215±0.178 | 1.241±0.166 | 0.942±0.475 | 0.772±0.417 | 2.466±0.181 | 2.465±0.180 | 0.703 | 0.908 | 0.606 |
| Frontal_Sup_Orb_R | 1.264±0.209 | 1.290±0.194 | 1.047±0.521 | 0.898±0.420 | 2.435±0.195 | 2.497±0.211 | 0.859 | 0.716 | 0.076 |
| Frontal_Mid_L | 1.161±0.189 | 1.119±0.219 | 0.923±0.551 | 0.721±0.523 | 2.401±0.116 | 2.394±0.116 | 0.271 | 0.788 | 0.323 |
| Frontal_Mid_R | 1.229±0.202 | 1.214±0.228 | 1.025±0.587 | 0.825±0.526 | 2.349±0.122 | 2.340±0.118 | 0.589 | 0.869 | 0.665 |
| Frontal_Mid_Orb_L | 1.122±0.156 | 1.129±0.166 | 0.901±0.428 | 0.739±0.376 | 2.637±0.197 | 2.619±0.189 | 0.966 | 0.707 | 0.915 |
| Frontal_Mid_Orb_R | 1.128±0.174 | 1.137±0.168 | 0.941±0.483 | 0.776±0.379 | 2.586±0.185 | 2.591±0.174 | 0.972 | 0.795 | 0.507 |
| Frontal_Inf_Oper_L | 1.225±0.191 | 1.219±0.215 | 1.057±0.475 | 0.920±0.434 | 2.533±0.109 | 2.514±0.117 | 0.460 | 0.343 | 0.977 |
| Frontal_Inf_Oper_R | 1.274±0.198 | 1.295±0.225 | 1.099±0.554 | 0.962±0.460 | 2.510±0.116 | 2.485±0.114 | 0.893 | 0.328 | 0.676 |
| Frontal_Inf_Tri_L | 1.247±0.186 | 1.234±0.204 | 1.048±0.501 | 0.901±0.444 | 2.382±0.119 | 2.376±0.112 | 0.503 | 0.288 | 0.301 |
| Frontal_Inf_Tri_R | 1.262±0.179 | 1.271±0.199 | 1.072±0.533 | 0.936±0.442 | 2.393±0.126 | 2.378±0.114 | 0.841 | 0.222 | 0.730 |
| Frontal_Inf_Orb_L | 1.237±0.196 | 1.262±0.184 | 0.973±0.438 | 0.865±0.355 | 2.644±0.157 | 2.610±0.138 | 0.673 | 0.402 | 0.541 |
| Frontal_Inf_Orb_R | 1.246±0.184 | 1.264±0.170 | 0.981±0.465 | 0.878±0.352 | 2.648±0.163 | 2.638±0.143 | 0.880 | 0.325 | 0.506 |
| Rolandic_Oper_L | 1.264±0.185 | 1.288±0.198 | 1.141±0.452 | 1.048±0.383 | 2.561±0.143 | 2.544±0.142 | 0.975 | 0.148 | 0.315 |
| Rolandic_Oper_R | 1.286±0.191 | 1.317±0.189 | 1.173±0.507 | 1.057±0.381 | 2.576±0.143 | 2.543±0.138 | 0.934 | 0.393 | 0.937 |
| Supp_Motor_L | 1.104±0.261 | 1.116±0.277 | 0.896±0.504 | 0.777±0.460 | 2.736±0.166 | 2.682±0.158 | 0.476 | 0.592 | 0.494 |
| Supp_Motor_R | 1.138±0.259 | 1.126±0.269 | 0.943±0.508 | 0.785±0.450 | 2.705±0.166 | 2.661±0.149 | 0.277 | 0.637 | 0.730 |
| Olfactory_L | 1.369±0.237 | 1.390±0.241 | 0.908±0.490 | 0.754±0.394 | 2.109±0.117 | 2.089±0.130 | 0.785 | 0.708 | 0.678 |
| Olfactory_R | 1.389±0.263 | 1.395±0.248 | 1.002±0.529 | 0.811±0.390 | 1.949±0.162 | 1.956±0.170 | 0.523 | 0.225 | 0.804 |
| Frontal_Sup_Med_L | 1.189±0.285 | 1.206±0.299 | 0.994±0.615 | 0.850±0.539 | 2.729±0.146 | 2.665±0.136 | 0.649 | 0.474 | 0.165 |
| Frontal_Sup_Med_R | 1.141±0.297 | 1.158±0.300 | 0.983±0.616 | 0.796±0.544 | 2.690±0.135 | 2.680±0.165 | 0.851 | 0.770 | 0.743 |
| Frontal_Med_Orb_L | 1.347±0.196 | 1.385±0.209 | 1.188±0.563 | 1.025±0.497 | 2.461±0.179 | 2.415±0.153 | 0.831 | 0.564 | 0.307 |
| Frontal_Med_Orb_R | 1.358±0.217 | 1.405±0.225 | 1.230±0.597 | 1.061±0.494 | 2.459±0.173 | 2.446±0.166 | 0.689 | 0.984 | 0.658 |
| Rectus_L | 1.349±0.238 | 1.412±0.220 | 1.131±0.571 | 0.978±0.447 | 2.363±0.145 | 2.367±0.163 | 0.514 | 0.709 | 0.715 |
| Rectus_R | 1.411±0.256 | 1.469±0.251 | 1.229±0.614 | 1.068±0.465 | 2.310±0.193 | 2.362±0.196 | 0.624 | 0.896 | 0.274 |
| Insula_L | 1.275±0.184 | 1.297±0.197 | 0.952±0.448 | 0.858±0.390 | N/A | N/A | 0.962 | 0.196 | N/A |
| Insula_R | 1.291±0.189 | 1.306±0.193 | 0.957±0.475 | 0.851±0.366 | N/A | N/A | 0.791 | 0.457 | N/A |
| Cingulum_Ant_L | 1.333±0.218 | 1.354±0.230 | 1.128±0.631 | 0.966±0.535 | 2.509±0.158 | 2.477±0.176 | 0.653 | 0.583 | 0.536 |
| Cingulum_Ant_R | 1.345±0.240 | 1.377±0.244 | 1.162±0.649 | 0.954±0.543 | 2.206±0.130 | 2.202±0.126 | 0.841 | 0.520 | 0.961 |
| Cingulum_Mid_L | 1.302±0.187 | 1.313±0.193 | 1.157±0.563 | 0.967±0.496 | 2.374±0.117 | 2.360±0.118 | 0.594 | 0.936 | 0.575 |
| Cingulum_Mid_R | 1.337±0.211 | 1.348±0.197 | 1.180±0.575 | 0.950±0.508 | 2.258±0.128 | 2.241±0.140 | 0.597 | 0.366 | 0.867 |
| Cingulum_Post_L | 1.200±0.189 | 1.221±0.196 | 1.154±0.548 | 1.005±0.507 | 1.995±0.166 | 2.011±0.183 | 0.752 | 0.895 | 0.487 |
| Cingulum_Post_R | 1.142±0.207 | 1.137±0.200 | 1.080±0.517 | 0.948±0.476 | 1.477±0.132 | 1.461±0.147 | 0.529 | 0.731 | 0.852 |
| Hippocampus_L | 1.399±0.179 | 1.430±0.231 | 0.996±0.181 | 0.956±0.174 | N/A | N/A | 0.630 | 0.913 | N/A |
| Hippocampus_R | 1.420±0.188 | 1.434±0.246 | 1.029±0.201 | 0.967±0.159 | N/A | N/A | 0.971 | 0.341 | N/A |
| ParaHippo_L | 1.371±0.238 | 1.378±0.249 | 0.895±0.267 | 0.869±0.223 | 2.782±0.197 | 2.760±0.237 | 0.821 | 0.440 | 0.926 |
| ParaHippo_R | 1.369±0.267 | 1.350±0.244 | 0.915±0.293 | 0.846±0.220 | 2.803±0.173 | 2.753±0.250 | 0.445 | 0.663 | 0.387 |
| Amygdala_L | 1.497±0.262 | 1.508±0.277 | 0.911±0.255 | 0.885±0.249 | N/A | N/A | 0.723 | 0.455 | N/A |
| Amygdala_R | 1.543±0.292 | 1.537±0.289 | 0.912±0.306 | 0.840±0.223 | N/A | N/A | 0.497 | 0.469 | N/A |
| Calcarine_L | 1.273±0.211 | 1.295±0.202 | 0.929±0.632 | 0.796±0.457 | 1.936±0.109 | 1.948±0.103 | 0.743 | 0.632 | 0.231 |
| Calcarine_R | 1.227±0.206 | 1.250±0.196 | 0.776±0.602 | 0.704±0.442 | 1.932±0.125 | 1.931±0.110 | 0.778 | 0.542 | 0.368 |
| Cuneus_L | 1.325±0.193 | 1.349±0.195 | 1.017±0.537 | 0.896±0.417 | 2.105±0.132 | 2.103±0.126 | 0.838 | 0.966 | 0.359 |
| Cuneus_R | 1.346±0.206 | 1.363±0.211 | 1.002±0.533 | 0.902±0.439 | 2.090±0.126 | 2.082±0.122 | 0.556 | 0.517 | 0.298 |
| Lingual_L | 1.263±0.157 | 1.277±0.163 | 0.905±0.489 | 0.790±0.372 | 2.093±0.122 | 2.079±0.127 | 0.529 | 0.589 | 0.684 |
| Lingual_R | 1.215±0.179 | 1.228±0.172 | 0.782±0.437 | 0.705±0.332 | 2.101±0.121 | 2.088±0.119 | 0.541 | 0.921 | 0.881 |
| Occipital_Sup_L | 1.258±0.178 | 1.253±0.205 | 0.953±0.568 | 0.780±0.414 | 2.117±0.122 | 2.109±0.139 | 0.414 | 0.468 | 0.550 |
| Occipital_Sup_R | 1.283±0.231 | 1.301±0.195 | 0.965±0.556 | 0.839±0.439 | 2.123±0.139 | 2.111±0.139 | 0.823 | 0.723 | 0.410 |
| Occipital_Mid_L | 1.335±0.194 | 1.330±0.199 | 1.038±0.602 | 0.836±0.431 | 2.251±0.126 | 2.242±0.137 | 0.338 | 0.313 | 0.467 |
| Occipital_Mid_R | 1.343±0.244 | 1.353±0.199 | 1.000±0.574 | 0.850±0.443 | 2.254±0.118 | 2.258±0.141 | 0.608 | 0.883 | 0.104 |
| Occipital_Inf_L | 1.420±0.238 | 1.424±0.225 | 1.157±0.609 | 0.972±0.433 | 2.370±0.162 | 2.330±0.160 | 0.614 | 0.269 | 0.535 |
| Occipital_Inf_R | 1.343±0.270 | 1.345±0.227 | 1.060±0.512 | 0.918±0.406 | 2.395±0.181 | 2.354±0.194 | 0.409 | 0.471 | 0.957 |
| Fusiform_L | 1.349±0.149 | 1.380±0.204 | 1.054±0.364 | 0.952±0.280 | 2.704±0.121 | 2.673±0.139 | 0.672 | 0.751 | 0.620 |
| Fusiform_R | 1.300±0.157 | 1.304±0.163 | 0.975±0.331 | 0.880±0.258 | 2.661±0.125 | 2.617±0.181 | 0.779 | 0.748 | 0.525 |
| Postcentral_L | 1.030±0.173 | 1.028±0.203 | 0.805±0.418 | 0.685±0.423 | 2.189±0.137 | 2.156±0.126 | 0.975 | 0.484 | 0.799 |
| Postcentral_R | 1.060±0.172 | 1.074±0.219 | 0.847±0.476 | 0.693±0.429 | 2.113±0.139 | 2.092±0.133 | 0.810 | 0.953 | 0.564 |
| Parietal_Sup_L | 1.132±0.247 | 1.078±0.305 | 0.911±0.619 | 0.680±0.485 | 2.239±0.142 | 2.205±0.147 | 0.279 | 0.668 | 0.945 |
| Parietal_Sup_R | 1.088±0.247 | 1.076±0.269 | 0.878±0.604 | 0.657±0.499 | 2.231±0.157 | 2.196±0.145 | 0.991 | 0.840 | 0.894 |
| Parietal_Inf_L | 1.271±0.213 | 1.259±0.244 | 1.018±0.634 | 0.805±0.517 | 2.324±0.130 | 2.304±0.128 | 0.769 | 0.689 | 0.589 |
| Parietal_Inf_R | 1.268±0.218 | 1.283±0.237 | 1.061±0.660 | 0.831±0.528 | 2.273±0.143 | 2.251±0.129 | 0.760 | 0.955 | 0.526 |
| SupraMarginal_L | 1.354±0.195 | 1.372±0.207 | 1.164±0.549 | 0.992±0.460 | 2.526±0.140 | 2.506±0.139 | 0.722 | 0.479 | 0.656 |
| SupraMarginal_R | 1.360±0.197 | 1.396±0.196 | 1.145±0.599 | 0.968±0.473 | 2.456±0.131 | 2.447±0.132 | 0.426 | 0.641 | 0.255 |
| Angular_L | 1.408±0.206 | 1.423±0.286 | 1.126±0.650 | 0.948±0.521 | 2.390±0.155 | 2.398±0.147 | 0.783 | 0.339 | 0.081 |
| Angualr_R | 1.361±0.214 | 1.364±0.213 | 1.108±0.665 | 0.898±0.508 | 2.387±0.150 | 2.362±0.144 | 0.715 | 0.974 | 0.500 |
| Precuneus_L | 1.301±0.190 | 1.303±0.236 | 1.127±0.597 | 0.932±0.485 | 2.301±0.137 | 2.283±0.137 | 0.472 | 0.920 | 0.917 |
| Precuneus_R | 1.334±0.224 | 1.337±0.230 | 1.161±0.607 | 0.966±0.494 | 2.354±0.128 | 2.326±0.122 | 0.504 | 0.934 | 0.900 |
| Paracentral_Lob_L | 0.942±0.238 | 0.949±0.278 | 0.719±0.384 | 0.629±0.400 | 2.376±0.196 | 2.329±0.186 | 0.770 | 0.835 | 0.613 |
| Paracentral_Lob_R | 1.106±0.221 | 1.115±0.265 | 0.898±0.429 | 0.781±0.398 | 2.360±0.183 | 2.346±0.177 | 0.655 | 0.915 | 0.810 |
| Caudate_L | 1.259±0.214 | 1.195±0.215 | 0.696±0.329 | 0.610±0.288 | N/A | N/A | 0.207 | 0.846 | N/A |
| Caudate_R | 1.163±0.250 | 1.099±0.232 | 0.709±0.283 | 0.549±0.275 | N/A | N/A | 0.360 | 0.070 | N/A |
| Putamen_L | 1.611±0.208 | 1.624±0.243 | 0.944±0.293 | 0.849±0.260 | N/A | N/A | 0.437 | 0.953 | N/A |
| Putamen_R | 1.620±0.222 | 1.621±0.234 | 1.038±0.308 | 0.934±0.247 | N/A | N/A | 0.345 | 0.454 | N/A |
| Pallidum_L | 1.846±0.280 | 1.842±0.281 | 1.182±0.218 | 1.123±0.245 | N/A | N/A | 0.219 | 0.728 | N/A |
| Pallidum_R | 1.833±0.281 | 1.831±0.286 | 1.289±0.225 | 1.226±0.249 | N/A | N/A | 0.282 | 0.932 | N/A |
| Thalamus_L | 1.351±0.146 | 1.322±0.164 | 1.491±0.163 | 1.474±0.200 | N/A | N/A | 0.129 | 0.882 | N/A |
| Thalamus_R | 1.372±0.183 | 1.326±0.167 | 1.387±0.161 | 1.347±0.204 | N/A | N/A | 0.076 | 0.503 | N/A |
| Heschl_L | 1.217±0.185 | 1.230±0.199 | 1.007±0.497 | 0.905±0.401 | 2.435±0.162 | 2.411±0.169 | 0.895 | 0.172 | 0.536 |
| Heschl_R | 1.204±0.190 | 1.222±0.186 | 1.015±0.551 | 0.876±0.384 | 2.395±0.180 | 2.350±0.177 | 0.879 | 0.695 | 0.951 |
| Temporal_Sup_L | 1.300±0.186 | 1.332±0.220 | 1.106±0.505 | 0.978±0.411 | 2.575±0.149 | 2.545±0.141 | 0.631 | 0.237 | 0.630 |
| Temporal_Sup_R | 1.317±0.185 | 1.358±0.189 | 1.142±0.520 | 1.008±0.400 | 2.564±0.144 | 2.540±0.148 | 0.474 | 0.372 | 0.431 |
| Templ_Pole_Sup_L | 1.221±0.250 | 1.273±0.292 | 0.834±0.404 | 0.809±0.335 | 3.266±0.220 | 3.159±0.248 | 0.600 | 0.109 | 0.083 |
| Templ_Pole_Sup_R | 1.204±0.234 | 1.254±0.224 | 0.835±0.419 | 0.782±0.319 | 3.032±0.209 | 2.970±0.257 | 0.488 | 0.312 | 0.576 |
| Temporal_Mid_L | 1.364±0.177 | 1.397±0.240 | 1.094±0.507 | 0.942±0.409 | 2.584±0.125 | 2.557±0.118 | 0.600 | 0.801 | 0.877 |
| Temporal_Mid_R | 1.415±0.199 | 1.448±0.206 | 1.135±0.545 | 0.980±0.417 | 2.577±0.126 | 2.558±0.130 | 0.718 | 0.920 | 0.588 |
| Templ_Pole_Mid_L | 1.235±0.213 | 1.298±0.298 | 0.879±0.346 | 0.872±0.330 | 3.456±0.219 | 3.352±0.271 | 0.400 | 0.131 | 0.236 |
| Templ_Pole_Mid_R | 1.187±0.178 | 1.236±0.251 | 0.844±0.317 | 0.785±0.280 | 3.235±0.206 | 3.170±0.254 | 0.272 | 0.601 | 0.250 |
| Temporal_Inf_L | 1.405±0.212 | 1.460±0.301 | 1.082±0.463 | 0.963±0.386 | 2.769±0.142 | 2.718±0.149 | 0.439 | 0.858 | 0.279 |
| Temporal_Inf_R | 1.410±0.224 | 1.435±0.237 | 1.097±0.477 | 0.949±0.361 | 2.751±0.140 | 2.719±0.204 | 0.700 | 0.606 | 0.690 |

*Abbreviations: APOE4* apolipoprotein epsilon 4
