# Supplementary material for: Correlations between APOE4 allele and regional amyloid and tau burdens in cognitively normal older individuals
Source: Sci Rep. 2022 Aug 22;12:14307. doi: 10.1038/s41598-022-18325-2 (PMC9395408; doi:10.1038/s41598-022-18325-2)
Supplement: Supplementary file 4 — Supplementary Table 3. [file 41598_2022_18325_MOESM4_ESM.docx]

**Supplementary table 3** Regional amyloid, tau, and cortical thickness in APOE4 carriers and APOE4 non-carriers and their group differences after controlling for age and sex

| AAL ROIs | Tau | | Amyloid | Cortical thickness | |
| --- | --- | --- | --- | --- | --- |
|  | adjusted *p* | | adjusted *p* | adjusted *p* | |
| Precentral_L | 0.611 | | 0.076 | 0.191 | |
| Precentral_R | 0.878 | | 0.081 | 0.146 | |
| Frontal_Sup_L | 0.339 | | 0.035^*^(E4+>E4-) | 0.316 | |
| Frontal_Sup_R | 0.524 | | 0.035^*^(E4+>E4-) | 0.418 | |
| Frontal_Sup_Orb_L | 0.338 | | 0.018^*^(E4+>E4-) | 0.952 | |
| Frontal_Sup_Orb_R | 0.425 | | 0.047^*^(E4+>E4-) | 0.067 | |
| Frontal_Mid_L | 0.224 | | 0.021^*^(E4+>E4-) | 0.692 | |
| Frontal_Mid_R | 0.678 | | 0.027^*^(E4+>E4-) | 0.647 | |
| Frontal_Mid_Orb_L | 0.791 | | 0.013^*^(E4+>E4-) | 0.568 | |
| Frontal_Mid_Orb_R | 0.744 | | 0.016^*^(E4+>E4-) | 0.877 | |
| Frontal_Inf_Oper_L | 0.871 | | 0.063 | 0.313 | |
| Frontal_Inf_Oper_R | 0.569 | | 0.090 | 0.198 | |
| Frontal_Inf_Tri_L | 0.682 | | 0.054 | 0.763 | |
| Frontal_Inf_Tri_R | 0.764 | | 0.082 | 0.439 | |
| Frontal_Inf_Orb_L | 0.419 | | 0.087 | 0.142 | |
| Frontal_Inf_Orb_R | 0.534 | | 0.110 | 0.695 | |
| Rolandic_Oper_L | 0.465 | | 0.161 | 0.475 | |
| Rolandic_Oper_R | 0.325 | | 0.096 | 0.157 | |
| Supp_Motor_L | 0.789 | | 0.129 | 0.062 | |
| Supp_Motor_R | 0.788 | | 0.041^*^(E4+>E4-) | 0.085 | |
| Olfactory_L | 0.594 | | 0.029^*^(E4+>E4-) | 0.334 | |
| Olfactory_R | 0.882 | | 0.009^*^(E4+>E4-) | 0.791 | |
| Frontal_Sup_Med_L | 0.713 | | 0.121 | 0.006^*^(E4+>E4-) | |
| Frontal_Sup_Med_R | 0.729 | | 0.046^*^(E4+>E4-) | 0.691 | |
| Frontal_Med_Orb_L | 0.263 | | 0.057 | 0.082 | |
| Frontal_Med_Orb_R | 0.198 | | 0.052 | 0.645 | |
| Rectus_L | 0.094 | | 0.058 | 0.870 | |
| Rectus_R | 0.162 | | 0.058 | 0.104 | |
| Insula_L | 0.474 | | 0.161 | N/A | |
| Insula_R | 0.637 | | 0.109 | N/A | |
| Cingulum_Ant_L | 0.570 | | 0.084 | 0.249 | |
| Cingulum_Ant_R | 0.425 | | 0.030^*^(E4+>E4-) | 0.864 | |
| Cingulum_Mid_L | 0.740 | | 0.026^*^(E4+>E4-) | 0.489 | |
| Cingulum_Mid_R | 0.758 | | 0.009^*^(E4+>E4-) | 0.427 | |
| Cingulum_Post_L | 0.500 | | 0.082 | 0.573 | |
| Cingulum_Post_R | 0.882 | | 0.103 | 0.489 | |
| Hippocampus_L | 0.377 | | 0.167 | N/A | |
| Hippocampus_R | 0.700 | | 0.029^*^(E4+>E4-) | N/A | |
| ParaHippo_L | 0.859 | | 0.517 | 0.553 | |
| ParaHippo_R | 0.636 | | 0.090 | 0.185 | |
| Amygdala_L | 0.808 | | 0.528 | N/A | |
| Amygdala_R | 0.897 | | 0.080 | N/A | |
| Calcarine_L | 0.500 | | 0.120 | 0.519 | |
| Calcarine_R | 0.474 | | 0.379 | 0.981 | |
| Cuneus_L | 0.456 | | 0.107 | 0.909 | |
| Cuneus_R | 0.635 | | 0.198 | 0.684 | |
| Lingual_L | 0.612 | | 0.093 | 0.520 | |
| Lingual_R | 0.651 | | 0.206 | 0.524 | |
| Occipital_Sup_L | 0.882 | | 0.025^*^(E4+>E4-) | 0.713 | |
| Occipital_Sup_R | 0.582 | | 0.109 | 0.591 | |
| Occipital_Mid_L | 0.867 | | 0.013^*^(E4+>E4-) | 0.688 | |
| Occipital_Mid_R | 0.769 | | 0.062 | 0.849 | |
| Occipital_Inf_L | 0.914 | | 0.024^*^(E4+>E4-) | 0.126 | |
| Occipital_Inf_R | 0.972 | | 0.052 | 0.185 | |
| Fusiform_L | 0.320 | | 0.047^*^(E4+>E4-) | 0.164 | |
| Fusiform_R | 0.892 | | 0.043^*^(E4+>E4-) | 0.105 | |
| Postcentral_L | 0.948 | | 0.082 | 0.116 | |
| Postcentral_R | 0.686 | | 0.036^*^(E4+>E4-) | 0.344 | |
| Parietal_Sup_L | 0.253 | | 0.009^*^(E4+>E4-) | 0.167 | |
| Parietal_Sup_R | 0.786 | | 0.013^*^(E4+>E4-) | 0.158 | |
| Parietal_Inf_L | 0.754 | | 0.021^*^(E4+>E4-) | 0.348 | |
| Parietal_Inf_R | 0.685 | | 0.015^*^(E4+>E4-) | 0.317 | |
| SupraMarginal_L | 0.597 | | 0.033^*^(E4+>E4-) | 0.387 | |
| SupraMarginal_R | 0.258 | | 0.038^*^(E4+>E4-) | 0.672 | |
| Angular_L | 0.730 | | 0.056 | 0.744 | |
| Angualr_R | 0.918 | | 0.024^*^(E4+>E4-) | 0.289 | |
| Precuneus_L | 0.953 | | 0.024^*^(E4+>E4-) | 0.409 | |
| Precuneus_R | 0.946 | | 0.026^*^(E4+>E4-) | 0.165 | |
| Paracentral_Lob_L | 0.875 | | 0.168 | 0.138 | |
| Paracentral_Lob_R | 0.820 | | 0.080 | 0.644 | |
| Caudate_L | 0.069 | | 0.083 | N/A | |
| Caudate_R | 0.106 | | 0.001^*^(E4+>E4-) | N/A | |
| Putamen_L | 0.725 | | 0.034^*^(E4+>E4-) | N/A | |
| Putamen_R | 0.977 | | 0.018^*^(E4+>E4-) | N/A | |
| Pallidum_L | 0.940 | | 0.128 | N/A | |
| Pallidum_R | 0.974 | | 0.114 | N/A | |
| Thalamus_L | 0.256 | | 0.576 | N/A | |
| Thalamus_R | 0.107 | | 0.200 | N/A | |
| Heschl_L | 0.679 | | 0.154 | 0.373 | |
| Heschl_R | 0.568 | | 0.056 | 0.121 | |
| Temporal_Sup_L | 0.363 | | 0.080 | 0.195 | |
| Temporal_Sup_R | 0.185 | | 0.065 | 0.324 | |
| Templ_Pole_Sup_L | 0.257 | | 0.666 | 0.007^*^(E4+>E4-) | |
| Templ_Pole_Sup_R | 0.182 | | 0.364 | 0.126 | |
| Temporal_Mid_L | 0.366 | | 0.038^*^(E4+>E4-) | 0.165 | |
| Temporal_Mid_R | 0.321 | | 0.042^*^(E4+>E4-) | 0.357 | |
| Templ_Pole_Mid_L | 0.157 | | 0.897 | 0.015^*^(E4+>E4-) | |
| Templ_Pole_Mid_R | 0.190 | | 0.218 | 0.099 | |
| Temporal_Inf_L | 0.219 | 0.081 | | | 0.037^*^(E4+>E4-) |
| Temporal_Inf_R | 0.508 | 0.027^*^(E4+>E4-) | | | 0.295 |

*Abbreviations: APOE4* apolipoprotein epsilon 4
