# Supplementary figures and images for: Correlations between APOE4 allele and regional amyloid and tau burdens in cognitively normal older individuals
Source: Sci Rep. 2022 Aug 22;12:14307. doi: 10.1038/s41598-022-18325-2 (PMC9395408; doi:10.1038/s41598-022-18325-2)

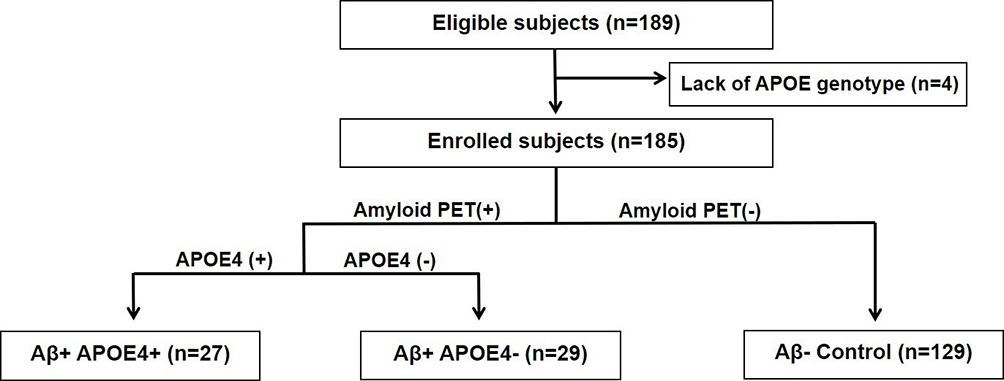

Supplement: Supplementary file 5 — Supplementary Figure 1. [file 41598_2022_18325_MOESM5_ESM.tif]

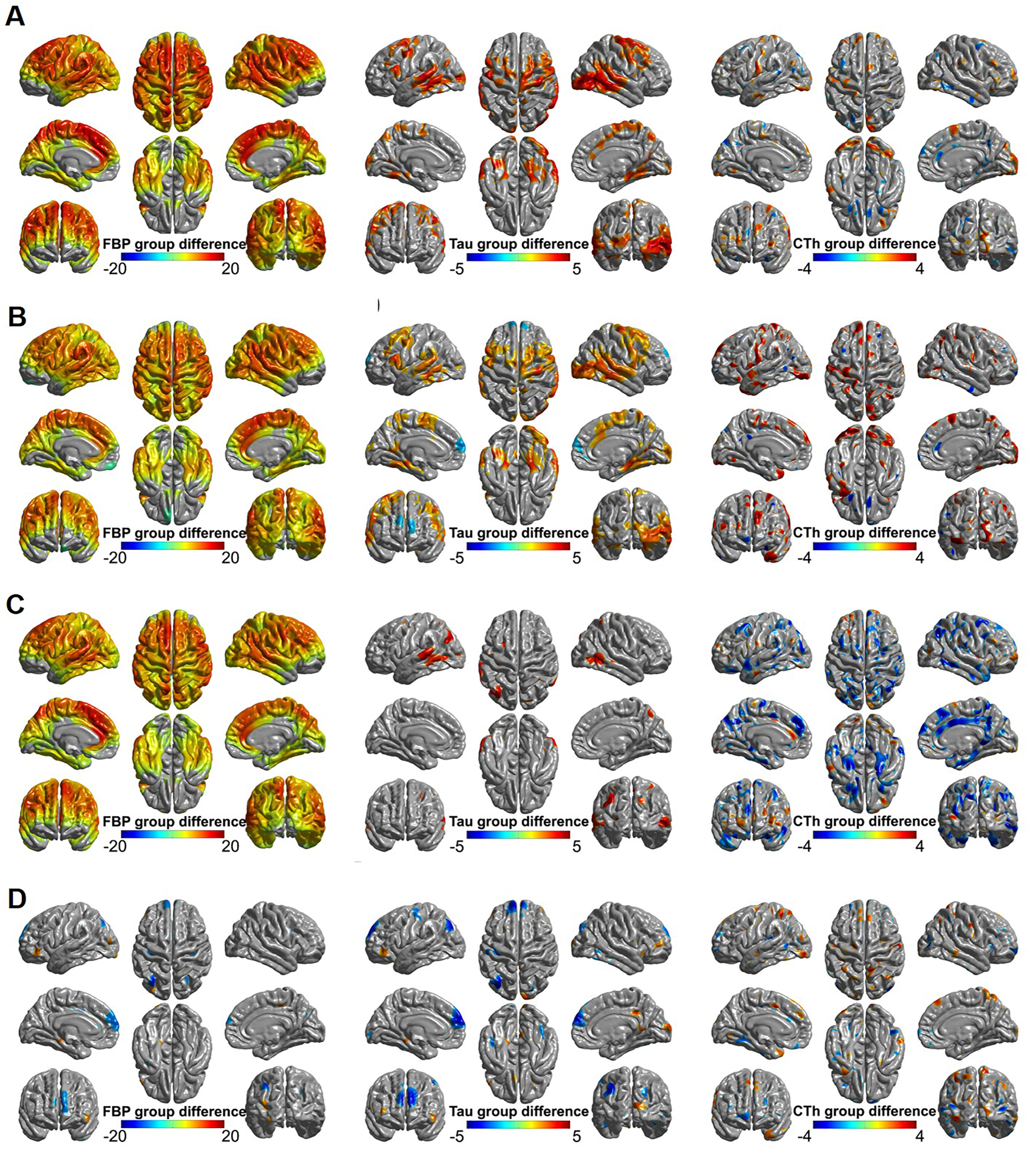

Supplement: Supplementary file 6 — Supplementary Figure 2. [file 41598_2022_18325_MOESM6_ESM.tif]
